# Delayed Stanford Type A Aortic Dissection After Endovascular Repair of Abdominal Aortic and Common Iliac Artery Aneurysms Resulting in Stent Graft Collapse: A Case Report

**DOI:** 10.7759/cureus.57707

**Published:** 2024-04-06

**Authors:** Domen Lah, Tomaž Cvetko, Silva Breznik

**Affiliations:** 1 Department of Radiology, Faculty of Medicine University of Maribor, Maribor, SVN; 2 Department of Radiology, University Medical Centre Maribor, Maribor, SVN

**Keywords:** abdominal aortic aneurysm, vascular interventional radiology-evar, endovascular stent graft, endovascular aneurysm repair, type a aortic dissection

## Abstract

We discuss a rare case of Stanford type A aortic dissection (TAAD) occurring several months after endovascular aneurysm repair (EVAR) of the abdominal aortic and the common iliac artery (CIA) aneurysms. The patient underwent urgent surgery for TAAD treatment but died on the table due to intractable bleeding. We conclude that TAAD was likely unrelated to the initial EVAR procedure but rather to atherosclerosis, hypertension, and prior aortic valve replacement. Only a few cases in the literature report TAAD and total collapse of the abdominal aortic stent graft.

## Introduction

An abdominal aortic aneurysm (AAA) is an abnormal permanent dilatation of the abdominal aorta by at least 150% compared to a relatively normal adjacent vessel diameter, which is approximately 2.0 cm below the origin of the renal arteries [1]. Therefore, a diameter of more than 3.0 cm is considered an infrarenal aortic aneurysm. The most common risk factors for AAA include advanced age (> 65 years), male sex, smoking, coronary artery disease, hypertension, previous myocardial infarction, peripheral arterial disease, and a family history of AAA, among others [2].

Since AAA is most often asymptomatic, it is usually discovered randomly with ultrasonography. Larger aneurysms can manifest as indigestion, lower limb or pelvis paresthesia, and symptoms due to pressure on the renal vessels, bile ducts, or ureters [3]. Computer tomography angiography (CTA) is normally used to determine the exact location, size, and involvement of other vessels. Most AAA are infrarenal and fusiform in shape, although they may also be located elsewhere and saccular in shape. Treatment with open surgical repair or endovascular aneurysm repair (EVAR) is indicated when AAA exceeds 5.0-5.5 cm in diameter and/or is rapidly expanding (> 0.5 cm over six months) and/or is symptomatic [1,4].

EVAR is associated with lower perioperative 30-day all-cause mortality as well as a significant reduction in perioperative morbidity when compared to open surgery. Complications following EVAR occur in 16-30% of cases and include endoleaks, endograft migration or collapse, kinking, and stenosis of an endograft limb, graft infection, end-organ ischemia, cerebrovascular and cardiovascular events, and post-implantation syndrome [5]. The occurrence of aortic dissection following EVAR is a rarely reported event in the current literature and is usually associated with excessive oversizing, use of devices with active fixation systems, injuries during the procedure, or a de novo dissection. It can lead to the collapse and occlusion of the previous endograft and is fatal in about 30% of the cases when aneurysm rupture occurs [6].

## Case presentation

**Figure 1:**
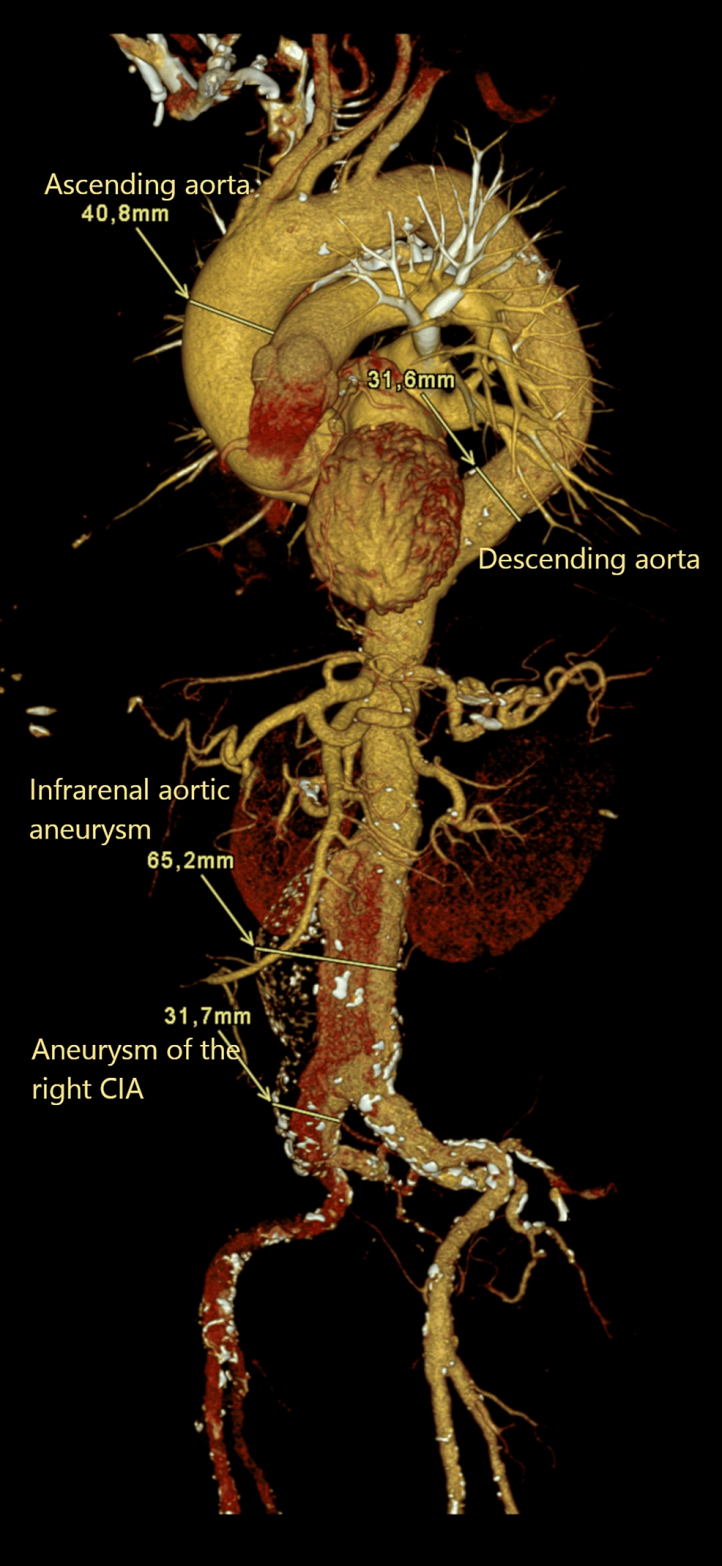
Computed tomography angiography of the initial aortic aneurysms, three-dimensional view.

A 64-year-old man with a history of systemic hypertension, chronic obstructive pulmonary disease, diabetes mellitus, chronic heart failure, prior aortic valve replacement surgery (with biological valve), and a known AAA was referred to the radiology department for elective EVAR. Results of the CTA showed a fusiform abdominal aortic aneurysm with a diameter of approximately 65 mm and length of 130 mm with extension to both common iliac arteries (CIA), diameters right 32 mm, left 20 mm. The aneurysm extended over the iliac bifurcation (Figure 1).

**Figure 2:**
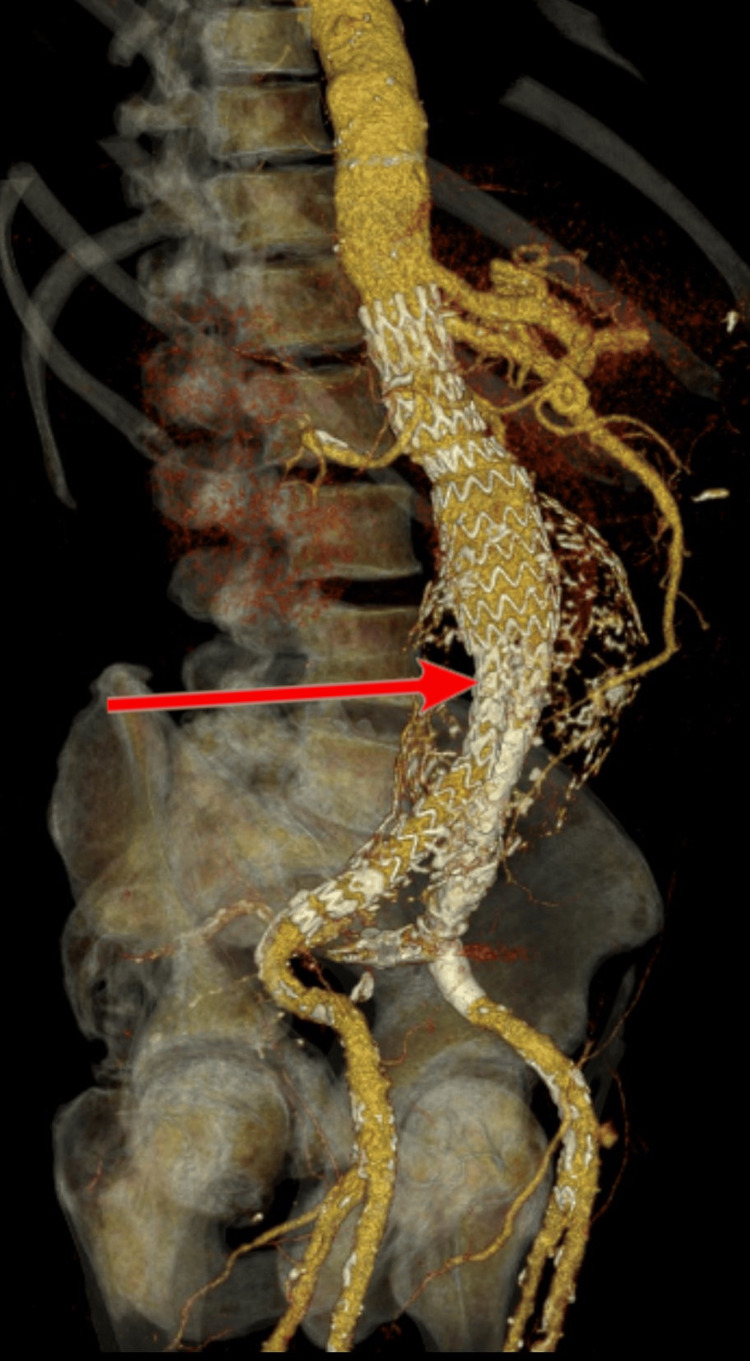
Computed tomography angiography control after endovascular aneurysm repair, stent graft position (arrow).

The endovascular treatment was performed under general anesthesia using the standard bilateral femoral artery approach. Two Perclose ProGlide™ sewing systems (Abbott Laboratories, Chicago, Illinois, United States) were placed bilaterally. A Zenith Alpha™ bifurcated main body graft (Cook Group Incorporated, Bloomington, Indiana, United States) with a diameter of 36 mm and a length of 128 mm was advanced through the right femoral artery. The main body of the graft was positioned below the renal arteries and expanded with a balloon catheter. On each side, Zenith Alpha Spiral-Z limb was deployed, a 13 mm x 110 mm graft through the right side introducer sheath and a 20 mm x 77 mm graft through the left side introducer sheath. A kissing balloon technique with 10 mm balloons was used at the graft bifurcation. The right internal iliac artery (IIA) was covered with the right limb to exclude the right CIA aneurysm. A completion angiogram showed patency of the graft system and a small type II endoleak at the level of the right IIA. The procedure was completed successfully without any visible post-procedural bleeding and the patient was hemodynamically stable. He was discharged home after two days and was prescribed dual antiplatelet therapy with acetylsalicylic acid 100 mg daily for life and clopidogrel 75 mg daily for three months. Figure 2 shows the final position of aortic stent graft after EVAR.

**Figure 3:**
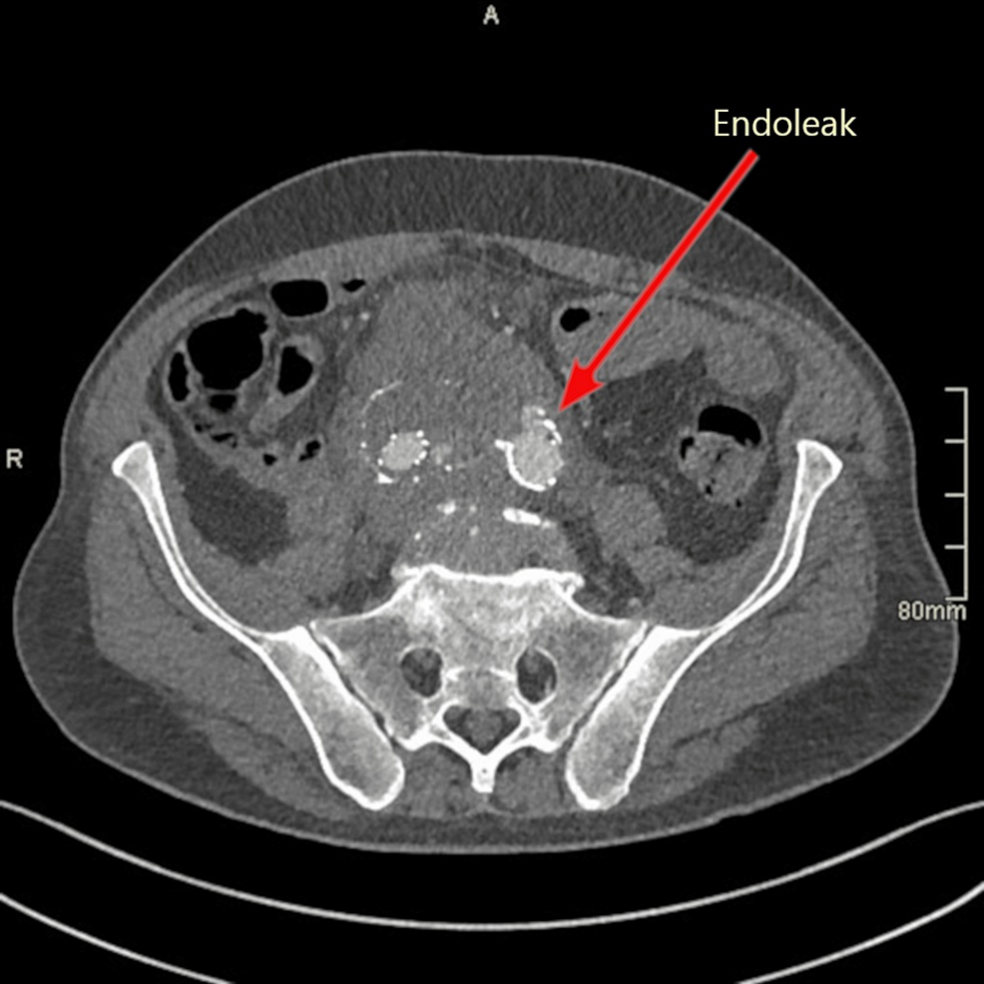
Type 3 endoleak into the aneurysmal sac of the left common iliac artery.

The follow-up CTA one month after the procedure showed an endoleak into the aneurysmal sac of the left CIA, which most likely originated from the left iliac limb (Figure 3). Fifty-five days after EVAR, the patient underwent emergency endovascular therapy with aortography, embolization of the left IIA, and stent graft placement over the left IIA ostium due to the rapid iliac aneurysm sac expansion on the right side. The procedure was performed under local anesthesia using the standard left femoral artery approach with a retrograde puncture and placement of a 6 French (Fr) introducer, which was later replaced with a 12 Fr introducer. A diagnostic catheter angiography showed a contrast leak from the left branch of the EVAR graft into the aneurysmal sac towards the right IIA (type 3 endoleak). The left IIA was canulated and embolized using a Concerto™ helix and 3D coil system (Medtronic plc, Dublin, Ireland) with 16 mm and 18 mm diameters. Next, a Viabahn® 13 mm x 100 mm stent graft (W. L. Gore & Associates, Inc., Newark, Delaware, United States) was placed at the level of the defect in the left limb of the previous graft. The pulling line got stuck during deployment and the graft did not cover the entire left IIA. The problem was solved by realigning the graft with a new Viabahn 11 mm x 100 mm graft and post dilatation of the junctions with a 12 mm balloon.

A completion angiogram showed patency of the graft system and no endoleak in the aneurysmal sac, but there was still a contrast leak between the 13 mm and 11 mm grafts into the left IIA. Therefore, the proximal branch of the graft was covered with another Zenith Alpha Spiral-Z ZISL 11-59 mm graft and post-dilatated with a 12 mm balloon. A final angiogram did not show any endoleaks and hemostasis was achieved with a dual ProGlide system. Furthermore, the patient also had a pseudoaneurysm of the common femoral artery. On the periprocedural follow-up CTA, perigraft fat tissue inflammation was also seen, suggesting a possible graft infection. The patient received linezolid, ciprofloxacin, and rifampin as antimicrobial therapy until the control positron emission tomography with CT (PET-CT) scan was performed. He was discharged home four days after the procedure and was prescribed dual antiplatelet therapy with clopidogrel for the next three months and acetylsalicylic acid for life, along with analgesic therapy with metamizole or paracetamol/tramadol. Ciprofloxacin and rifampin were prescribed for longer period due to confirmed infection of the aorta and periaortic tissue. 

**Figure 4:**
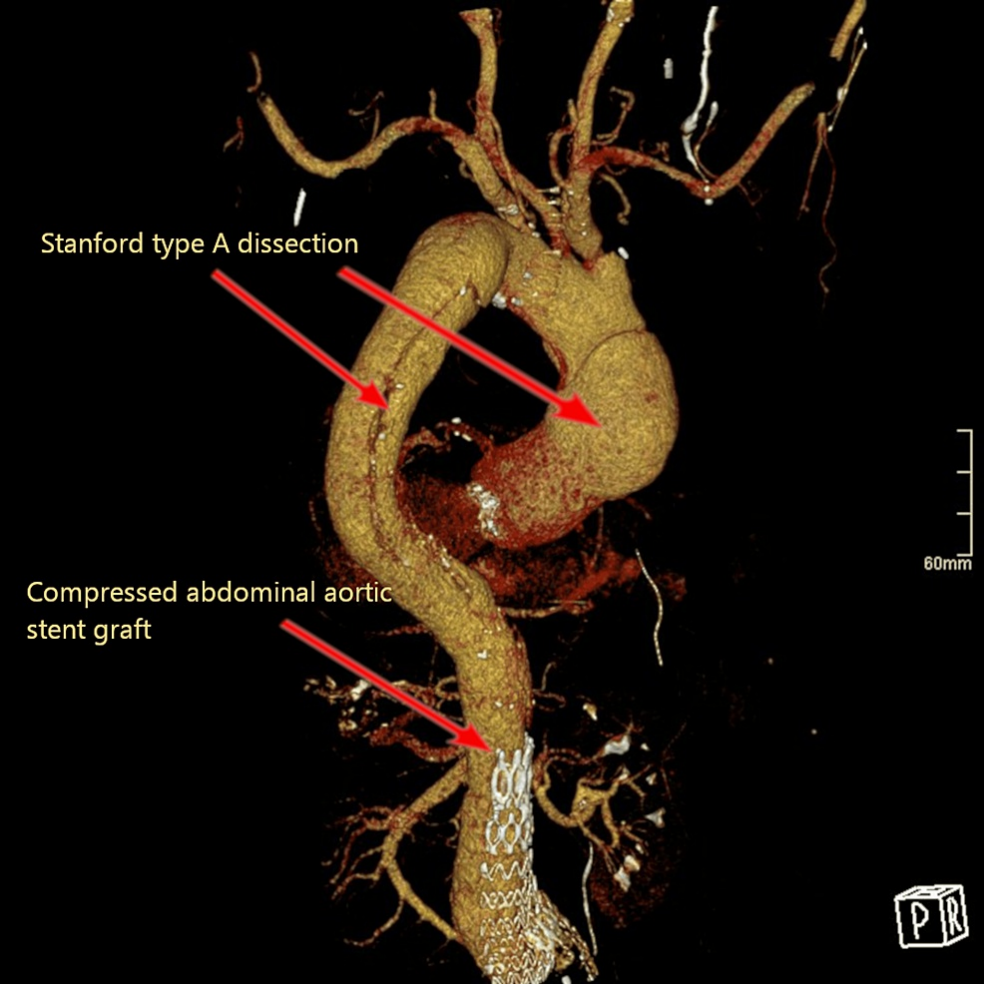
Stanford type A aortic dissection, three-dimensional view.

**Figure 5:**
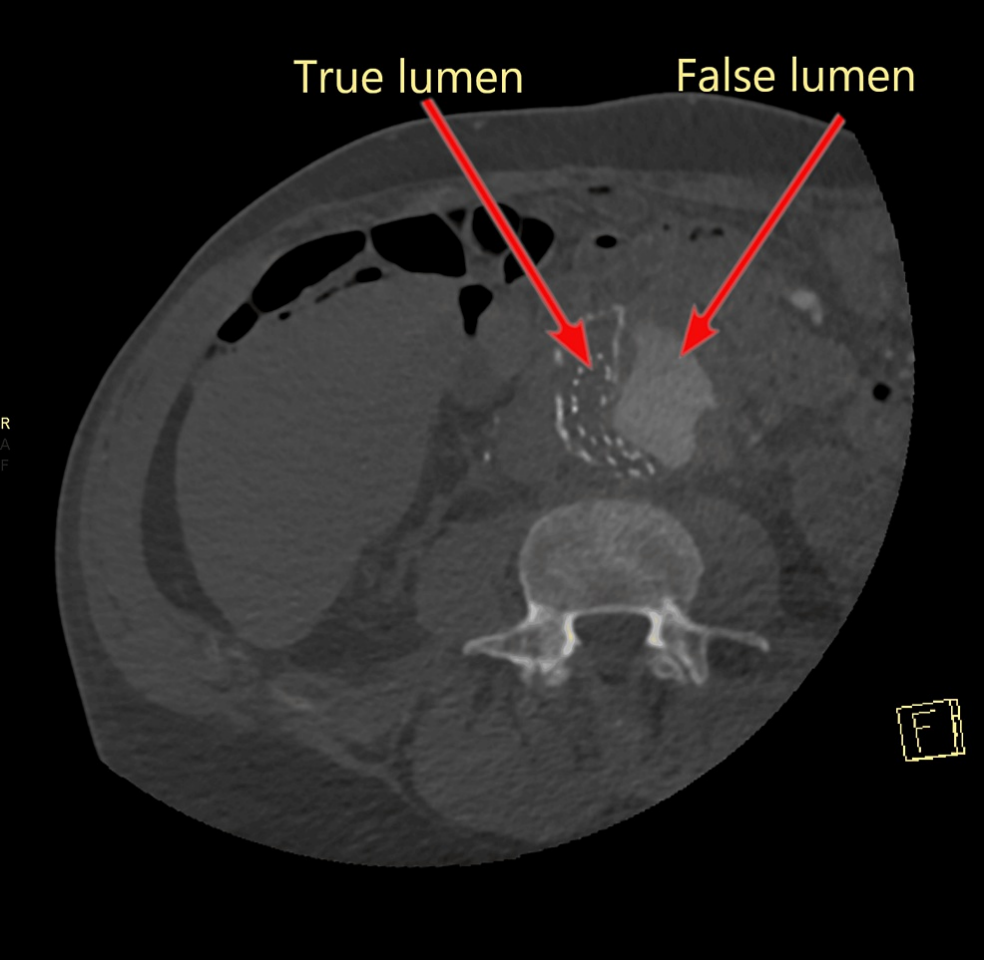
Stanford type A aortic dissection, false lumen compressing the aortic stent graft.

**Figure 6:**
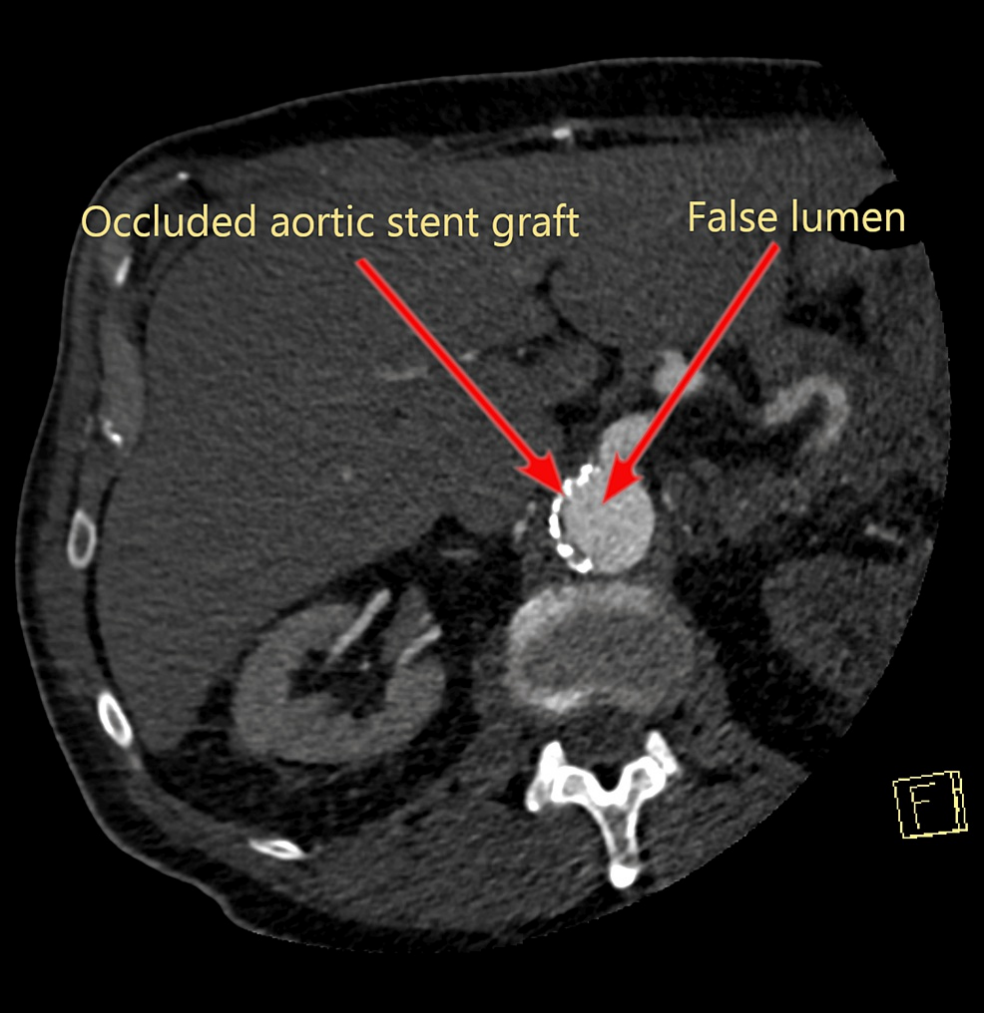
Complete occlusion of the aortic stent graft by the false lumen of Stanford type A aortic dissection.

Two hundred forty-two days after initial EVAR placement and 180 days after the endoleak repair, the patient presented to the emergency department complaining of chest, back, abdomen and right lower extremity pain, as well as cold right foot and ankle. He also had elevated blood pressure (218/123 mmHg) despite medication. An emergency CTA of the thoracoabdominal aorta showed a Stanford type A aortic dissection (TAAD) (Figure 4) starting from the artificial aortic valve and extending into the abdominal aorta with partial and complete occlusion due to compression of the EVAR graft (Figure 5, Figure 6) and occlusion of both stent graft limbs. The left renal artery was also occluded, and the left kidney was ischemic. The blood flow to both legs was through false lumen and reduced, but the celiac trunk and the superior mesenteric artery were both patent. The patient underwent urgent surgery for TAAD treatment but died on the table due to intractable bleeding.

## Discussion

We present a case study of a patient who developed Stanford TAAD eight months (242 days) after undergoing EVAR for abdominal aortic and right CIA aneurysms, and nine months (282 days) after aortic valve replacement surgery.

After undergoing EVAR, patients may experience mild or severe complications, including endoleaks (14.6-16.8%), migration (0.1%), graft limb occlusion (0.9-4.7%), stent graft infection (0.6%), and stent graft-induced new entry (SINE) (3.4%) [7]. In general, TAAD accounts for about 58-62% of all aortic dissections with a total case-fatality rate of about 73%. Some of the most common risk factors for TAAD include hypertension, increased age, smoking, Marfan syndrome, Ehlers-Danlos syndrome, vascular inflammatory diseases, and iatrogenic factors (coronary catheterization, aortic cross-clamping during cardiac surgery, arterial cannulation during cardiac surgery, heart valve procedures) [8]. Iatrogenic dissection accounts for 1-5% of all TAADs [8], from which approximately 43%, 40%, and 7% result from cardiac surgery, endovascular cardiac interventions, and aortic endovascular interventions, respectively [9]. Surgical treatment is usually centered on excision of the proximal intimal tear, replacement of the ascending aorta, and re-establishment of a dominant flow in the distal true lumen [10].

TAAD following EVAR is a rare and challenging problem, which can lead to severe consequences such as expansion or rupture of the aneurysm, or endograft collapse with malperfusion due to excessive pressure in the false lumen from the lack of outflow [11]. The majority of reported aortic dissections following EVAR are Stanford type B [12-14]. The most common treatment in these cases was thoracic EVAR (TEVAR), with which a false lumen is potentially thrombosed and its pressure is decreased. In a case similar to ours, TAAD was treated with TEVAR, but unsuccessfully, since it only prolonged the patient’s survival to open repair with endograft replacement, which is another treatment option [11]. Additionally, most data reports TAAD following TEVAR rather than abdominal EVAR, with a postprocedural TAAD incidence of about 6.8%. Possible etiology of such complications may include intimal tears from wire manipulation, endografts rubbing the aortic wall, repeated balloon remodeling, and oversized endografts [9].

In the present case, the etiology of the dissection was likely unrelated to the initial EVAR procedure as the dissection occurred eight months following the procedure and was probably associated with atherosclerosis, hypertension, and prior aortic valve replacement surgery. TAAD caused a total collapse of the true aortic lumen through a previously inserted abdominal aortic bifurcation graft, due to the radial force exerted by the false lumen, and subsequent malperfusion of abdominal organs and lower limbs [15]. Furthermore, our patient had undergone aortic valve replacement nine months prior to TAAD development. Although a rare event, aortic valve replacement may also be a contributing factor in TAAD development, with an incidence of 0.6%; it is more common (incidence of 27%) in patients who have had an aortic dilatation of more than 50 mm at the time of aortic valve replacement. It has a high mortality rate of 1-2% per hour for the first 24-48 hours [16]. Even though our patient underwent aortic valve replacement surgery, there have been some reports of late TAAD development also following transcatheter aortic valve replacement (TAVR) [17,18].

An additional contributing factor to aortic dissection development is elevated blood pressure. It is reported that 67.3-76.6% of patients who develop aortic dissection also have hypertension. Elevated blood pressure along with atherosclerosis is associated with dilatation of the ascending aorta, loss of elastic fibers in the aortic media, and unstable connections between each elastic lamina, resulting in aortic dissection before or after aortic aneurysm formation. It is estimated that aortic dissection is roughly three times more likely to develop in individuals with hypertension than in individuals without hypertension [19].

## Conclusions

TAAD is a rare but major complication following EVAR. We conclude that the etiology of the dissection in our case was likely unrelated to the initial EVAR as the dissection occurred several months after the procedure and was more likely associated with atherosclerosis, hypertension, and prior aortic valve replacement surgery. Physicians should be aware of this complication, as it is even more likely to occur in older patients with several comorbidities and prior history of heart procedures.
